# Hyperamylasemia Caused by the Hantaan Virus: A Retrospective Study of 101 Patients with Hemorrhagic Fever with Renal Syndrome in West China

**DOI:** 10.1155/2022/4942697

**Published:** 2022-06-25

**Authors:** Sumei Zhang, Lei Shi, Xiaoqian Jiang, Xiaoling Yang, Libo Wu, Chuan Wang, Fuchun Jing, Tianyan Chen

**Affiliations:** ^1^Department of Infectious Diseases, Baoji People's Hospital, Yan'an University, Baoji 721000, China; ^2^Department of Infectious Diseases, The First Affiliated Hospital, Xi'an Jiaotong University, Xi'an 710061, China

## Abstract

**Background:**

Hyperamylasemia (HA) is an inconspicuous manifestation of hemorrhagic fever with renal syndrome (HFRS) in Baoji city, West China. Hantaan virus (HTNV) is the only pathogen-caused HFRS in this region, but the knowledge about HA in the local HFRS patients has been limited. The aim of this study was to investigate the characteristics of HA and its predictive risk factors for doctors to engage in timely monitoring and dealing with the possible serious changes prewarned by HA in the early stages of the disease to improve the final outcome.

**Methods:**

All HFRS patients with and without HA (HA and nHA groups, respectively) were treated in Baoji People's Hospital. The clinical characteristics between the two groups were compared by Student's **t**-test or Chi-square test. The risk factors for prognosis were measured by the logistic regression analysis. The predictive effects of prognosis in clinical and laboratory parameters were analyzed by the receiver operating characteristic curves.

**Results:**

46.53% of the patients demonstrated HA, among which 71.7% were severe and critical types of HFRS, greater than that in the nHA group (19.57%, **P** < 0.001). The hospitalization day and the general incidence of acute pancreatitis (AP) were longer or greater in the HA group than in the nHA group (**P** < 0.01). Age and the time from the onset of the first symptom to the patient being admitted to hospital (*T*_OA_) were the predictive risk factors for HA. The best cut-off values were the age of 54 years and *T*_OA_ of 5.5 days.

**Conclusion:**

HTNV-induced HA is a common clinical presentation of HFRS patients in West China. It can increase the severity, the hospitalization days of patients, and the incidence of AP in HFRS. Age and *T*_OA_ constituted independent risk factors for HA caused by HTNV.

## 1. Introduction

Hyperamylasemia (HA) refers to the elevation of serum amylase (s-AMY) over the upper limit of normal value (<135 IU/L). As s-AMY is mainly derived from the pancreas and salivary glands, HA is often theoretically believed to be one of the major presentations of pancreatic-salivary diseases such as acute pancreatitis (AP), pancreatic trauma, parotitis, and salivary gland obstruction with calculi [1]. However, because of the complexity of amylase synthesis and clearance inside the human body, HA is not the only specific laboratory item necessary to diagnose the above-mentioned diseases [2]. It can also be seen in clinical situations such as malignant tumors [3], renal failure [4], liver failure [5], intestinal diseases [6], female reproductive organ diseases [7], acidosis [8], and secondary to the use of some chemotherapy drugs [9]. In COVID-19 pandemic caused by the severe acute respiratory syndrome coronavirus 2, researchers have found HA accompanied by pancreatic injury [10].

Hantaan virus (HTNV) is one of the Hantaviruses of Bunyavirales order, Hantaviridae family. It is mainly carried by rodents and transmitted to humans through aerosolized particles of their excrement or via parasitic bites. There are four main known kinds of Hantaviruses: HTNV, Seoul, Dobrava-Belgrade, and Puumala (PUUV) viruses. All of them have pantropic virulence and can lead to hemorrhagic fever with renal syndrome (HFRS). As the earliest discovered member of this family, HTNV spreads in most areas of China, but in Shaanxi province of West China, it is the only pathogen-causing HFRS in recent decades [11].

HFRS is an infectious disease existing around the world, especially in Asian and European countries [12]. Its primary clinical manifestations include febrile, hypotensive, oliguric, diuretic, and convalescent stages. Patients with severe or critical pathological changes often die of systemic bleeding and multiple organ dysfunction syndromes [13]. Doctors now have the ability to give more advanced therapy to patients, such as continuous renal replacement therapy, but due to its complex clinical presentations, diverse complications, and scarcity of specific medicine, the mortality of HFRS is still over 10% [14]. As one of the fatal diseases threatening human health, HFRS can damage the kidney, heart, brain, lung, gastrointestinal organs, and blood vessels [15] and lead to sophisticated pathophysiological changes, including bleeding, elevation of blood urea nitrogen, and abnormalities of hepatic function [16, 17].

Previous studies have reported cases of HFRS with AP induced by different Hantaviruses [18–21], but no research focused on the characteristics of HA in HFRS caused by HTNV in Baoji city, Shaanxi province of West China. The reason behind of this is that s-AMY is not a routine laboratory test item in clinical practice of HFRS, and only those patients who have exact abdominal pain will be advised to measure s-AMY. Therefore, the present study retrospectively collected and analyzed the data related to HA from 101 HFRS patients who were admitted to the Baoji People's Hospital affiliated to Yan'an University. The results may help doctors in this region engage in timely monitoring and dealing with the possible serious changes prewarned by HA in the early stages of the disease to improve the final outcome.

## 2. Materials and Methods

### 2.1. Data Collection

A trained doctor was assigned in charge of the data collection. Data collected mainly included the following: (1) general demographics (sex and age); (2) clinical manifestations (symptoms, signs, iconography, and laboratory changes in different clinical stages); (3) vital timeline and levels of s-AMY: the time from the onset of the first symptom to the patient being admitted to hospital (*T*_OA_), the time from the onset of the first symptom to the patient being discharged (*T*_Discharge_), the hospitalization days (or hospital stay) of the patient (*T*_Hospitalization_), the time s-AMY began to increase (*T*_Initial_), the time s-AMY reached to peak (*T*_Peak_), the concentration of s-AMY starting to elevate (*C*_I−AMY_), the concentration of s-AMY reaching to peak (*C*_P−AMY_), and the concentration of s-AMY at the time point when the patients were discharged (*C*_D−AMY_); and (4) comorbidities, such as chronic obstructive pulmonary disease, hypertension, coronary heart disease, diabetes, liver disease, and lacunar infarction.

### 2.2. Study Population

**Figure 1:**
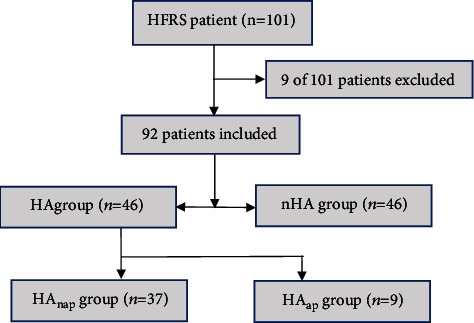
Grouping of the patients.

All patients with confirmed HFRS who were admitted to the Baoji People's Hospital affiliated to Yan'an University from January 2018 to December 2020 were enrolled in this study. They were divided into two groups: nonhyperamylasemia (nHA) and hyperamylasemia (HA) on the basis of the s-AMY level. To investigate the influence of HA on HFRS patients with and without AP, the patients in the HA group were further divided into two subgroups: HA without AP (HAnap subgroup, 37 cases) and HA with AP (HAap subgroup, 9 cases). The *T*_OA_, *T*_Discharge_, *T*_Hospitalization_, *T*_Initial_, *T*_Peak_, *C*_I−AMY_, *C*_P−AMY_, and *C*_D−AMY_ were carefully compared between these two subgroups. The risk factors related to HA were also statistically analyzed. The study was approved by the Ethics Committee of Baoji People's Hospital (ethics approval No: 202012001). Informed consent was waived due to the retrospective nature of the study. All patient data were anonymized. The grouping of patients is shown in Figure 1.

### 2.3. Clinical Types and Criteria for Diagnosis and Discharge

The diagnosis and inclusion criteria of HFRS patients were those who had a laboratory confirmation by the detection of specific serum IgM and IgG antibodies to HTNV using the method of enzyme-linked immunosorbent assay (ELISA) [22], and those patients who were diagnosed with HFRS but did not finish their treatment were excluded.

The definition of HA was made by a level of s-AMY over 135 IU/L.

For AP diagnosis, the guidelines of the International Association of Pancreatology were followed [23, 24]. The detail was that the patients met at least two of the following three criteria: (1) clinical (acute persistent, severe mid-upper abdominal pain often radiates to the back); (2) laboratory (serum AMY or lipase > 3× upper limit of normal); and/or (3) imaging (CT, MRI, or ultrasonography found characteristic changes of AP).

According to the previously published study [25], the clinical types and severity of HFRS were classified into four kinds: (1) mild refers to a patient having acute kidney injury (AKI) with no oliguria and hypotension; (2) moderate refers to a patient having uremia, hypotension, effusion in the bulbar conjunctiva, hemorrhage in the skin and mucous membranes, and AKI with oliguria; (3) severe refers to a patient having severe uremia, effusion in bulbar conjunctiva and either peritoneum or pleura, hemorrhage in the skin and mucous membranes, hypotension, and AKI with oliguria (urine output of 50–500 mL/day) for less than 5 days or anuria (urine output of <100 mL/day) for less than 2 days; and (4) critical refers to a patient having more than one of the following complications: refractory shock lasting over 2 days, visceral hemorrhage, heart failure, pulmonary edema, brain edema, severe secondary infection, and severe AKI with oliguria over 5 days or anuria complications over 2 days.

All patients were discharged from the hospital when they met the following criteria: (1) most clinical symptoms disappeared; (2) urine volume recovered to normal level; (3) most laboratory tests became normal; and (4) patients could live independently without needing assistance.

### 2.4. Statistical Analysis

All data collected were processed using the SPSS 22.0 software. Normally distributed continuous variables were expressed as the mean ± SD and analyzed by Student's *t*-test for comparisons. Nonnormally distributed variables were compared using the nonparametric Mann–Whitney *U*-test. Categorical variables were presented as frequencies and percentages, and the Chi-square test was used to evaluate the differences. A value of *P* < 0.05 was considered significant. Correlations between demographic characteristics, clinical manifestations, laboratory values, and HA were measured by the Cox multiple regression. A binary logistic regression analysis was used to identify clinical risk factors for prognosis. Predicting values for the prognosis was performed with the laboratory parameters that were tested with receiver operating characteristic (ROC) curves and quantified by calculating the area under the curve (AUC) and the 95% confidence interval (CI).

## 3. Results

### 3.1. General Characteristics of the Patients and the Incidence of HA

A total of 101 HFRS patients during the study period were enrolled. After exclusion of 9 patients due to waiving life-supporting therapies or accidental death, 92 patients (mean age 50.7 ± 17.2, 76 men (82.6%)) were included in the final analysis. In group nHA, there were 39 males (aged 10-72 years, average 45.28 ± 15.21 years) and 7 females (aged 18-59 years, average 36.29 ± 14.94 years), while in group HA, there were 37 males (aged 18-81 years, average 56.84 ± 16.15 years) and 9 females (aged 29-82 years, average 60.11 ± 17.61 years), respectively.

Thirty-three patients had comorbidities (some of them having more than one such diseases), mainly including chronic obstructive pulmonary disease (*n* = 6, 18.2%; 1 in the nHA group, and 5 in the HA group), hypertension (*n* = 14, 42.4%; 6 in the nHA group, and 8 in the HA group), coronary heart disease (*n* = 6, 18.2%; 2 in the nHA group, and 4 in the HA group), diabetes (*n* = 1, 3.0%; in the HA group), liver disease (*n* = 9, 27.3%; 4 in the nHA group, and 5 in the HA group), and lacunar infarction (*n* = 11, 33.3%; 3 in the nHA group, and 8 in the HA group). All of the comorbidities demonstrated no statistical difference between these two groups (*P* > 0.05).

Of the 101 HFRS patients, 47 had HA, and the incidence of HA was 46.53%. At the time of admission, only the difference in abdominal distention was significant (*P* < 0.05), and other main gastrointestinal symptoms, including nausea, vomiting, upper abdominal pain, and diarrhea, were not significantly different between the nHA and HA groups (*P* > 0.05). The patients were discharged from the hospital after these symptoms all disappeared. Four patients had imaging changes of AP in the HA group, but no patient had findings consistent with AP in the nHA group (*P* < 0.05). The values of 8 laboratory parameters, neutrophil count, s-AMY, procalcitonin (PCT), *α*-hydroxybutyrate dehydrogenase (HBDH), creatine kinase isoenzyme MB (CK-MB), prothrombin time (PT), international normalized ratio (INR), and activated partial thromboplastin time (APTT) were higher in the HA group than in the nHA group (*P* < 0.05 and *P* < 0.01, respectively). However, at the time of discharge, 10 items remained significantly different between the two groups (*P* < 0.05 and *P* < 0.01, respectively). The count of white blood cell (WBCs), lymphocyte, red blood cell (RBCs), platelet (PLTs), and the values for hemoglobin (HB), alanine aminotransferase (ALT) and HBDH were higher, but the levels of s-AMY, blood urea nitrogen (UR), and blood creatine (CR) were lower in the HA group than in the nHA group. Although the symptoms of all patients disappeared by the time of discharge, there were still 33 (71.7%) patients in the HA group whose s-AMY levels did not recover to normal ranges (Table 1).

### 3.2. Clinical Features of HFRS Patients with and without HA

As shown in Table 2, the general incidence of AP was 8.91% in the 101 patients with HFRS; in the HA group, this number was 19.57%, but in the nHA group, it was 0 (*P* < 0.01). A total of 71.7% of the patients in the HA group were identified as severe and critical types, but in the nHA group, the patients mainly presented as mild and moderate types, and the severe and critical types accounted for only 19.57%. There was a significant difference between the two groups (*P* < 0.001), indicating that HA was associated with high incidence of AP and the severity of HFRS.

**Figure 2:**
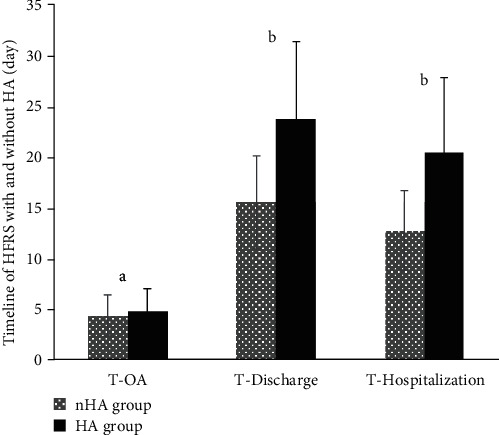
Timeline of HFRS patients with and without HA (day, mean ± SD). Compared between nHA and HA group, a means *P* > 0.05, and b refers to *P* < 0.001.

Compared with the nHA group, the *T*_OA_ of patients in the HA group was 4.70 ± 2.24 days, which was slightly longer than that in the former group (4.28 ± 1.97 days), but no significant difference was found (*P* > 0.05). However, the *T*_Discharge_ of patients in the HA group was far longer than that in the nHA group (23.67 ± 7.68 vs. 15.46 ± 4.56 days, *P* < 0.001). The same phenomenon also existed in the *T*_Hospitalization_ between the two groups (HA group vs. nHA group, 20.28 ± 7.51 vs. 12.67 ± 3.94 days, *P* < 0.001), meaning that HA may increase the HFRS patients' hospitalization days (Figure 2).

### 3.3. Influence of HA on HFRS Patients with and without AP

**Figure 3:**
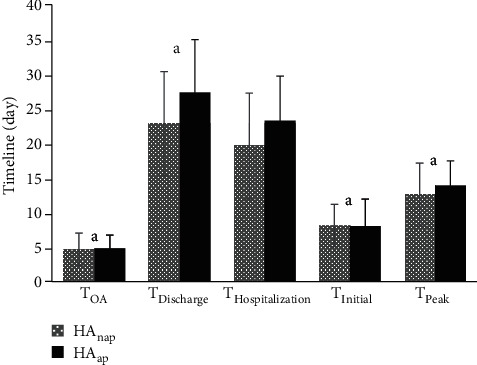
Timeline (day, mean ± SD) in the HAnap and HAap subgroups. Compared between HAnap and HAap subgroups, a means *P* > 0.05.

**Figure 4:**
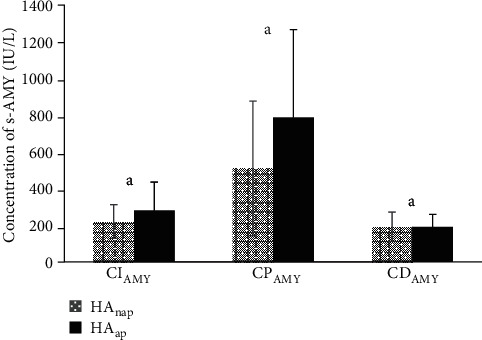
Concentration of s-AMY (IU/L, mean ± SD) in the HAnap and HAap subgroups. Compared between HAnap and HAap subgroups, a means *P* > 0.05.

The influence of HA on HFRS patients with and without AP were analyzed by the timeline of clinical progress and concentration changes of s-AMY in the HAnap and HAap subgroups. Interestingly, the results showed that the *T*_OA_, *T*_Discharge_, *T*_Hospitalization_, *T*_Initial_, and *T*_Peak_ between both groups did not demonstrate significant differences (*P* > 0.05). Compared with the patients without AP, though the patients with AP had greater values of the *C*_I−AMY_ and *C*_P−AMY_, there was also no significant difference between the two groups with regard to them and the *C*_D−AMY_ (*P* > 0.05) (Figures 3 and 4).

### 3.4. Pearson's Correlation and Logistic Regression Analysis of the Risk Factors for HA

Due to the important influence of HA on the severity and hospitalization day of HFRS patients caused by HTNV, we further analyzed the correlation and risk factors for HA.

Of the items we measured by multiple linear regression, age, RBC, HB, UR, CR, and INR were directly correlated with HA, among which RBC and HB were negatively correlated with HA (*P* < 0.001), but the remaining parameters were positively correlated with HA (*P* < 0.05, *P* < 0.001) (Table 3).

Further analysis using a binary logistic regression showed that age, *T*_OA_ and thrombin time (TT) were independent risk factors for HA. Their OR values were 1.697, 6.145, and 4.254, with 95% CIs of 1.165-2.471, 2.851-13.247, and 1.371-13.199 (*P* < 0.01, *P* < 0.001, and *P* < 0.05), respectively. Regression coefficients of the three factors (Table 4) were used to calculate a logit of HA as follows: the logarithm of odds of AMY = −8.607 + 0.529Age + 1.816*T*_OA_ + 1.448*TT*.

### 3.5. ROC and the Predictive Values of the Risk Factors for HA

**Figure 5:**
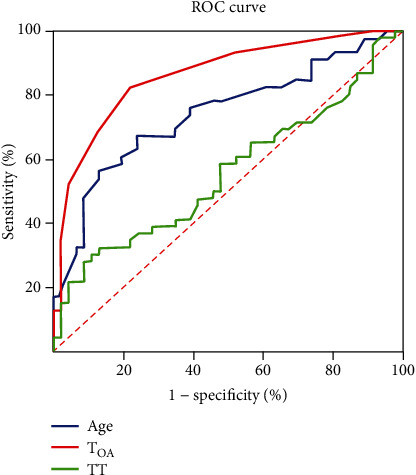
ROC analysis of the risk factors age, *T*_OA_, and *TT* for predicting value to HA. The results showed that age and *T*_OA_ were statistically significant (*P* < 0.001) for predicting whether serum AMY would elevate during the clinical course of HFRS.

To explore the predictive value of the risk factors for HA, ROC curves and AUCs were calculated (Table 5 and Figure 5). The results showed that age and *T*_OA_ were statistically significant for predicting whether s-AMY would be elevated during the clinical course of HFRS (*P* < 0.001). The best cut-off values were age of 54 years and *T*_OA_ of 5.5 days.

## 4. Discussion

Although this was a retrospective study, it was the first attempt to systemically analyze the clinical features of HA in HFRS patients caused by HTNV in the Shaanxi Province, West China. Generally, most clinicians know that HA is closely related to pancreatitis and parotitis, but few of them notice it can happen in several other diseases. In fact, in addition to being secreted from the pancreas and salivary glands, s-AMY can also be produced by other tissues, such as skeletal muscle, fat, the small intestine, and the ovary [26], which means that injury to these tissues can elevate the level of s-AMY. HTNV, one of the major Hantaviruses, has pantropic virulence to different tissues of the human body and can damage almost all the important organs. This implies that HTNV not only leads to the most common presentation of HFRS and AKI but also induces pathological lesions in vascular endothelial cells, myocardial cells, hepatocytes, gastrointestinal mucosal cells, and alveolar epithelial cells [27], further resulting in diverse and complicated clinical manifestations, especially HA. Although previous documents reported that few HFRS patients were easily complicated with AP [18–21], whether HTNV infection could inevitably result in the occurrence of HA in these patients is still unknown. In this retrospective study, we reported a 46.53% incidence of HA in HFRS patients, a very high rate that is often not focused on, meaning that getting the s-AMY value earlier was quite necessary to the diagnosis, treatment, and prognosis of HFRS caused by HTNV.

Because HA is often related to AP but not limited to it, we were interested in the influence of HA on HFRS patients with and without AP. We found that a previous study analyzed HFRS complicated with AP in the Anhui Province of mid China showed that the incidence of AP in local HFRS patients was 8.43% [20], but in Albania, another country with a high incidence of HFRS, some researchers reported it was 11.1% [18]. In our study, this figure was 8.91%, which was similar to the former but not in accordance with the latter. We inferred that this was probably due to the difference in the Hantavirus serotypes, socioeconomic situation, climatic factors, contact rate between rodents and humans, and the hygiene status between different regions. In the Anhui Province, the main serotypes of Hantaviruses were HTNV and the Seoul virus [20]; in Albania, it was the Dobrava-Belgrade virus [18], but in the Shaanxi Province (including Baoji city), it was HTNV only [11]. This means that compared with other Hantaviruses, HTNV may lead to a similar incidence of AP in west China as in mid China. Even so, our results still implied that the incidence of AP in the HA group was obviously higher than that in the nHA group, indicating that the elevation of s-AMY was an important signal of AP in HFRS patients caused by HTNV. However, an important problem was that not all HTNV-induced HFRS patients with HA developed AP during the evolution of the disease. The pathogenic mechanisms which underlie this finding are still unclear. Meanwhile, in this study we found that after the patients in the HA group were separated into the HAnap and HAap subgroups, the *T*_OA_, *T*_Discharge_, *T*_Hospitalization_, *T*_Initial_, *T*_Peak_, *C*_I−AMY_, *C*_P−AMY_, and *C*_D−AMY_ between the two subgroups demonstrated no significant differences, which interested us to develop more research for answers.

The severity of HFRS usually reflects its prognosis. There were many factors that influence it. Du and colleagues found that high WBC counts, prolonged PT and APTT complicated with acute respiratory distress syndrome, cardiac failure, and encephalopathy were related to a prolonged hospital stay and high mortality of HFRS patients [25]. Our study revealed that another important factor, HA, was a vital factor that was associated with severe and critical patients (the ratio of severe and critical types of HFRS was 71.74% in the HA group but only 19.57% in the nHA group). This demonstrated that the complex HA in HFRS patients was derived partly from damage to the pancreas or salivary glands and partly from damage to other tissues by HTNV, and HA was positively correlated with the severity of HFRS once it occurred.

Hospitalization days or hospital stay (*T*_Hospitalization_) is usually in accordance with the seriousness and complexity of a disease. Briefly, the more serious a disease is, the longer the time a patient spends in the hospital. In our study, there was no significant difference in *T*_OA_ between the nHA and HA groups, but compared with the nHA group, the *T*_Discharge_ and *T*_Hospitalization_ in the HA group were obviously prolonged, demonstrating that HA could increase the hospital stay time and postpone the recovery period of HFRS patients. This was similar to the characteristics of HFRS in the HA group because the patients in this group were usually severe and critical cases and had complications such as multiple organ injury.

As seen in our results, HA was associated with high incidence of AP, the severity, and the prolonged hospitalization day of HFRS patients caused by HTNV. Unfortunately, s-AMY is not a routine laboratory test item in clinical practice of HFRS, which means misdiagnosis, missed diagnosis, late diagnosis, or poor prewarning of the disease. Therefore, despite other factors, earlier finding and predicting HA may be beneficial to the prevention, treatment, and prognosis of HTNV-caused HFRS patients and acquire a good outcome with less severity and complications.

Based on these findings, we further analyzed the possible factors causing HA after HTNV infection by multiple linear regression. The results showed that the patients' age, blood UR content, and INR were positively correlated with HA, while the peripheral blood count of RBCs and the level of HB were negatively correlated with HA, which were actually the known characteristics of HFRS in these patients. In addition, a binary logistic regression analysis demonstrated that the independent risk factors for HA in HFRS patients caused by HTNV were age, *T*_OA_, and TT. But only age and *T*_OA_ had the predictive value for HA as risk factors according to ROC curves and AUCs calculated. Thus, doctors should pay much attention to these factors to predict the emergence of HA.

As to the general characteristics of HA caused by HTNV, our study measured some clinical parameters. The results showed that the number of patients with abdominal distention in the nHA group was greater than that in the HA group, but the imaging changes of AP was mainly existing in the HA group. The laboratory parameters of the two groups presented more complex results. On the one hand, the patients in the HA group were found to have elevations of 8 factors (WBC, s-AMY, PCT, *α*-HBDH, CK-MB, PT, INR, and APTT) on admission compared to those in the nHA group. On the other hand, after the clinical symptoms in both groups disappeared at the discharge timepoint, the values of WBC, lymphocyte, RBC, HB, PLT, ALT, and *α*-HBDH were higher in the nHA group than in the HA group. Furthermore, a more interesting phenomenon was that the values of s-AMY, UR, and CR in the HA group were statistically higher than those in the nHA group at the time of discharge from the hospital. This implied that the influence of HTNV on different tissues, organs, and laboratory parameters was different in its clinical stages, especially at the level of s-AMY. It did not return to normal levels in 71.7% of patients in the HA group.

## 5. Conclusions

In conclusion, HTNV-induced HA is one of the common clinical presentations of HFRS patients in West China. It can increase the incidence of AP, the severity, and the hospitalization day of HFRS patients. Age, RBC, HB, UR, CR, and INR were directly correlated with the emergence of HA. There were three factors, age, *T*_OA_, and *TT*, constituting independent risk factors for HA, but only age and *T*_OA_ had predictive value to it in HFRS patients caused by HTNV. Timely monitoring HA and dealing with the possible serious changes prewarned by HA in the early stages of HFRS may greatly improve the final outcome of this disease.

## Figures and Tables

**Table 1:** General characteristics of the patients in the nHA and HA groups (*n*/mean ± SD).

Variable	On admission			On discharge		
	nHA	HA	*P* value	nHA	HA	*P* value
Chief symptoms						
Abdominal pain	3	9	>0.05	0	0	—
Nausea	39	34	>0.05	0	0	—
Vomiting	22	21	>0.05	0	0	—
Diarrhea	12	8	>0.05	0	0	—
Abdominal distension	15	6	<0.05	0	0	—
Imaging positive (CT/MRI/ultrasonic)	0	4	<0.05	0	0	—
Laboratory						
WBCs (×10^9^/L)	10.74 ± 6.14	12.31 ± 11.26	>0.05	6.82 ± 1.79	6.01 ± 1.78	<0.05
N (×10^9^/L)	5.69 ± 3.23	8.38 ± 8.40	<0.05	3.85 ± 1.32	3.75 ± 1.52	>0.05
L (×10^9^/L)	3.04 ± 2.42	2.57 ± 2.50	>0.05	2.13 ± 0.82	1.51 ± 0.59	<0.001
RBCs (×10^12^/L)	4.61 ± 0.64	4.73 ± 0.69	>0.05	3.87 ± 0.42	3.40 ± 0.69	<0.001
HB (g/L)	141.54 ± 20.72	148.17 ± 19.09	>0.05	117.65 ± 13.24	106.84 ± 19.93	<0.001
PLTs (×10^9^/L)	61.74 ± 39.32	51.72 ± 37.38	>0.05	266.23 ± 93.34	224.22 ± 88.18	<0.05
ALT (IU/L)	216.42 ± 1052.42	67.53 ± 86.59	>0.05	59.42 ± 34.24	41.37 ± 33.95	<0.05
AST (IU/L)	100.47 ± 126.43	136.08 ± 149.22	>0.05	38.58 ± 19.44	37.66 ± 15.50	>0.05
TBIL (*μ*mol/L)	13.43 ± 12.92	12.28 ± 5.29	>0.05	15.58 ± 7.07	16.85 ± 8.27	>0.05
DBIL (*μ*mol/L)	5.94 ± 7.43	5.72 ± 2.76	>0.05	6.10 ± 2.91	6.93 ± 3.00	>0.05
ALB (g/L)	32.77 ± 5.10	32.10 ± 5.06	>0.05	37.33 ± 4.58	36.02 ± 3.64	>0.05
AMY (U/L)	65.12 ± 27.68	112.60 ± 83.48	<0.001	120.45 ± 65.12	194.38 ± 76.45	<0.001
CRP (ng/L)	34.94 ± 25.42	39.45 ± 25.84	>0.05	3.70 ± 3.57	3.04 ± 4.64	>0.05
PCT (pg/L)	2.48 ± 4.12	8.53 ± 17.99	<0.05	0.13 ± 0.08	0.12 ± 0.08	>0.05
UR (mmol/L)	11.94 ± 8.264	16.104 ± 11.96	>0.05	5.00 ± 2.09	6.76 ± 3.68	<0.05
CR (*μ*mol/L)	200.11 ± 170.23	238.80 ± 246.01	>0.05	71.10 ± 27.04	86.87 ± 41.20	<0.05
*α*-HBDH (U/L)	405.21 ± 199.39	500.31 ± 213.98	<0.05	212.79 ± 42.16	164.97 ± 39.66	<0.001
CK (U/L)	199.48 ± 295.87	271.42 ± 334.15	>0.05	60.5 ± 58.09	64.03 ± 71.15	>0.05
CK-MB (U/L)	26.88 ± 17.14	38.85 ± 23.92	<0.05	9.52 ± 3.72	8.45 ± 4.64	>0.05
PT (second)	13.35 ± 1.17	14.02 ± 1.61	<0.05	12.42 ± 1.10	13.02 ± 5.21	>0.05
TT (second)	22.09 ± 16.18	31.20 ± 33.87	>0.05	17.80 ± 2.73	17.09 ± 1.87	>0.05
INR	1.02 ± 0.18	1.15 ± 0.35	<0.05	0.95 ± 0.11	1.03 ± 0.61	>0.05
APTT (second)	48.73 ± 16.07	57.19 ± 20.14	<0.05	37.32 ± 9.09	37.43 ± 7.90	>0.05
FGB (g/L)	3.27 ± 0.86	5.36 ± 16.44	>0.05	3.62 ± 0.97	3.76 ± 0.99	>0.05
D-Dimer (*μ*g/mL)	5.67 ± 5.13	5.40 ± 5.65	>0.05	3.12 ± 2.14	2.59 ± 2.60	>0.05

**Table 2:** Incidence of AP and severity of HFRS between patients with and without HA.

Group	Incidence of AP (*n* (%))				Clinical type/severity (*n* (%))					
	AP	No AP	*χ* ^2^	*P* value	Mild	Moderate	Severe	Critical	*χ* ^2^	*P* value
nHA	0 (0.00)	46 (100.00)	9.98	<0.001	7 (15.21)	30 (65.22)	9 (19.57)	0 (0.00)	31.29	<0.001
HA	9 (19.57)	37 (80.43)			0 (0.00)	13 (28.26)	19 (41.30)	14 (30.44)		

**Table 3:** Pearson's correlation analysis.

Parameter	*r*	*P* value
Age	0.28	<0.001
RBC	-0.33	<0.001
HB	-0.35	<0.001
UR	0.45	<0.001
CR	0.340	<0.001
INR	0.21	<0.05

**Table 4:** Independent risk factors for HA in HFRS patients.

Parameter	*B*	SEM	Wald	df	*P*	OR	95% CI for OR	
							Lower	Upper
Age	0.53	0.19	7.59	1	<0.001	1.69	1.17	2.47
*T*_OA_	1.82	0.39	21.47	1	<0.001	6.15	2.85	13.25
TT	1.45	0.58	6.28	1	<0.05	4.25	1.37	13.20
Constant	-8.61	1.84	21.96	1	<0.001			

**Table 5:** Predictive value for the risk factors on HA in patient with HFRS.

Parameter	AUC	*P* value	Cut-off value	Sensitivity	Specificity	95% CI for AUC	
						Lower	Upper
Age	0.74	<0.001	54.00	67.40	76.10	0.64	0.85
*T*_OA_	0.87	<0.001	5.50	82.60	78.30	0.79	0.94
*TT*	0.56	>0.05	24.80	32.60	87.00	0.44	0.68

## Data Availability

The original contributions presented in the study are included in the article, and further inquiries can be directed to the corresponding author.

## Abbreviations

HA: Hyperamylasemia
HFRS: Hemorrhagic fever with renal syndrome
HTNV: Hantaan virus
nHA: Nonhyperamylasemia
ROC: Receiver operating characteristic curves
HAnap: HA without AP
HAap: HA with AP
AP: Acute pancreatitis
CP: Chronic pancreatitis
RBC: Red blood cell
HB: Hemoglobin
s-AMY: Serum amylase
BUN: Blood urea nitrogen
CR: Blood creatine
INR: International normalized ratio
T_OA_: Time from the onset of the first symptom to the patient being admitted to hospital
PCT: Procalcitonin
HBDH: *α*-Hydroxybutyrate dehydrogenase
CK-MB: Creatine kinase isoenzyme MB
PT: Prothrombin time
APTT: Activated partial thromboplastin time
WBC: White blood cell
PLT: Platelet
ALT: Alanine aminotransferase
*T*_Discharge_: Time from the onset of the first symptom to the patient being discharged
*T*_Hospitalization_: Hospitalization days (or hospital stay) of the patient
*T*_Initial_: Time s-AMY beginning to elevate
*T*_Peak_: Time s-AMY reaching to peak
*C*_I-AMY_: Concentration of s-AMY starting to elevate
*C*_P-AMY_: Concentration of s-AMY reaching to peak
*C*_D-AMY_: Concentration of s-AMY of the patients while they were discharged
ELISA: Enzyme-linked immunosorbent assay
AKI: Acute kidney injury
AUC: Area under the curve
CI: 95% confidence interval
